# Supplementary figures and images for: Genome-wide association study stratified by MAPT haplotypes identifies potential novel loci in Parkinson’s disease
Source: medRxiv. 2023 Apr 15:2023.04.14.23288478. Preprint. [Version 1] doi: 10.1101/2023.04.14.23288478 (PMC10246147; doi:10.1101/2023.04.14.23288478)

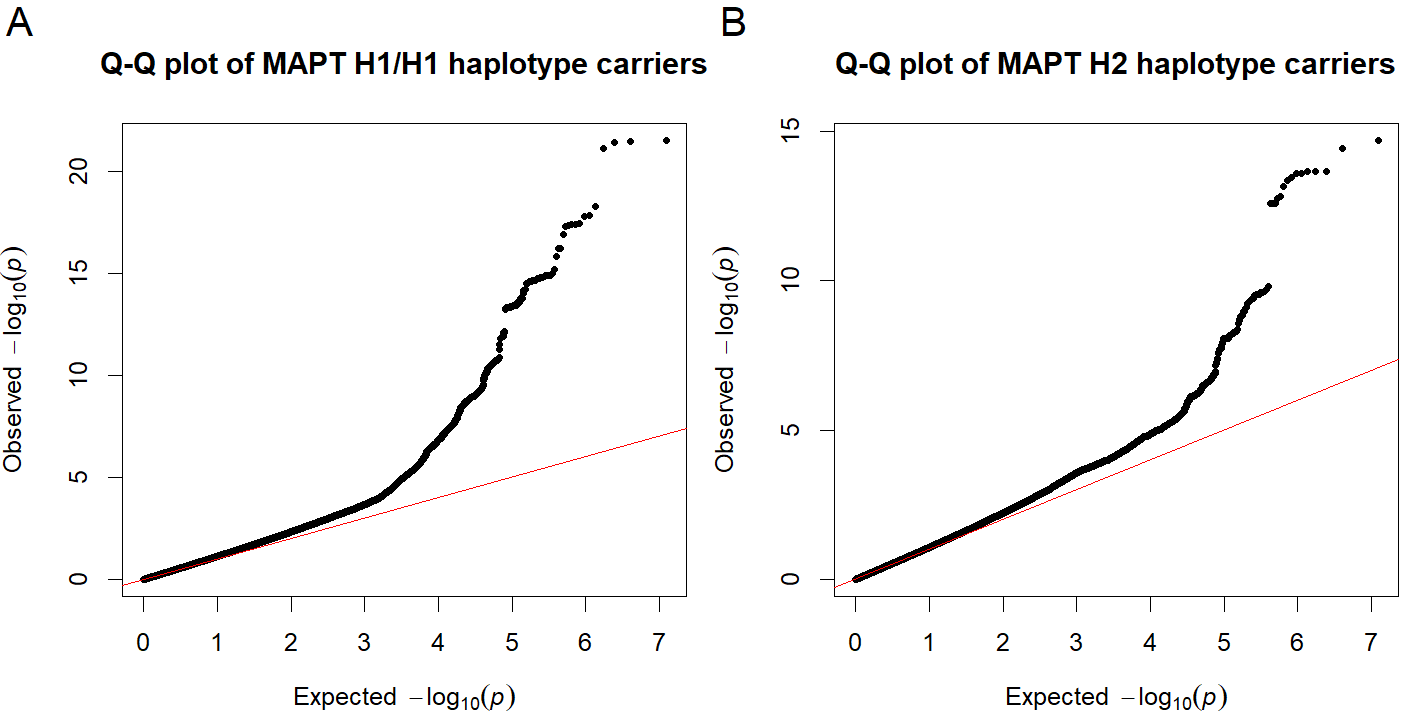

Supplement: Supplement 2 — Supplementary Table 1. Demography of studied population. Supplementary Table 2. Effect of novel SNPs EMP1 (rs56312722) and VANGL1 (rs11590278) variants on Parkinson’s disease age-at-onset in MAPT stratified cohorts. Supplementary Table 3. Burden analysis of rare EMP1 and VANGL1 variants in MAPT stratified cohorts. Supplementary Table 4. EMP1 variants with high CADD score. [file media-2.tif]
